# Prediction of missing common genes for disease pairs using network based module separation on incomplete human interactome

**DOI:** 10.1186/s12864-017-4272-7

**Published:** 2017-12-06

**Authors:** Pakeeza Akram, Li Liao

**Affiliations:** Department of Computer & Information Sciences, University of Delaware, Newark, DE USA

**Keywords:** Disease module separation, Optimization, Interactome, Missing gene, Comorbidity

## Abstract

**Background:**

Identification of common genes associated with comorbid diseases can be critical in understanding their pathobiological mechanism. This work presents a novel method to predict missing common genes associated with a disease pair. Searching for missing common genes is formulated as an optimization problem to minimize network based module separation from two subgraphs produced by mapping genes associated with disease onto the interactome.

**Results:**

Using cross validation on more than 600 disease pairs, our method achieves significantly higher average receiver operating characteristic ROC Score of 0.95 compared to a baseline ROC score 0.60 using randomized data.

**Conclusion:**

Missing common genes prediction is aimed to complete gene set associated with comorbid disease for better understanding of biological intervention. It will also be useful for gene targeted therapeutics related to comorbid diseases. This method can be further considered for prediction of missing edges to complete the subgraph associated with disease pair.

## Background

Genetic cause for diseases is complex and complicated, and can rarely be attributed to a single gene. Instead, often, multiple factors are involved in manifestation of disease symptoms. Furthermore, as genes can take on more than one function and different pathways and processes are intertwined and can crosstalk to one another, it is therefore also quite common that one gene may be implicated in two or more diseases. As a result, it is sensible and informative to examine not only the associated genes of one disease to understand its pathology but also the overlap between the sets of associated genes of two diseases of high comorbidity risk in order to shed lights on the interplay of the two diseases [1–3]. Yet, the knowledge that can be gained from a list of genes, or their product proteins, would be quite limited if not putting them in the biological context, such as the signaling transduction pathways, regulatory and metabolic pathways in which they are involved.

Numerous efforts are being taken to identify relationship between two diseases [4, 5]. Comorbidity refers to the phenomenon that two (or more) diseases co-occur. Bar the pure coincidence, comorbidity would indicate that the two diseases are somehow pathologically similar. The similarity may reveal at various levels: from more phenotyptic ones, such as disease symptoms or coexpression of associated genes, to more genotypic ones, such as sharing common genes between the respective gene sets associated with the diseases. Indeed, there are reports on disease relationship which incorporates the fact of common genetic origin of diseases [6, 7]. Recently emerged disease network theory has shifted focus to disease module [4]. Disease module contains those set of genes whose mutations have effect on phenotype, these set of genes are not scattered by chance in the interactome but they reside close to each other due to their interactions. These interactions form one or several connected subgraphs called as “disease module”. Specifically, network based separation of a disease pair A and B (SAB) is introduced to compare shortest distances between proteins within each disease to the shortest distances between disease pair A and B. Relationships between a pair of diseases that have been revealed via other means, such as gene ontology (GO) term similarity and relative risk (RR) for comorbidity, are to correlate with the overlapping of two disease modules, supporting the hypothesis: cause of disruption leading to one disease may cause another disease sharing common characteristics. For example, [4] used the disease history of 30 million individuals aged 65 and older (U.S. Medicare) to determine for each disease pair the relative risk RR of disease comorbidity, finding that the relative risk drops from RR ≥10 for S_AB_ < 0 to the random expectation of RR ≈ 1 for S_AB_ > 0. While the esults show great promise, however, a significant challenge presents due to the limitation of having very few data. For example, there are only 7% of the disease pairs which overlap with each other and have negative S_AB_ value. At the system level, only 20% of the disease interaction network has been captured [4].

In this study, we attempt to address the issue by a novel method to predict the missing genes in the disease module using available information such as genes association to diseases, relative risk of cooccurrence of two diseases and human interactome. Our work starts out with the findings about disease module separation S_AB_ from [4] and explores its utility as a powerful indicator to determine comorbid diseases: smaller SAB indicates that two selected diseases are more closely located in the interactome, and hence may show comorbid behavior. To complete the set of gene associated with disease and contribute towards completing the interactome, it is critical to identify missing common genes. The method formulates the task of searching for missing common genes as an optimization problem to minimize a network based module separation between two subgraphs formed by mapping the disease associated genes onto the interactome. Tested on a dataset of more than 600 disease pairs using cross-validation, it is shown that the method achieves an average ROC score of 0.95.

## Methods

In this section, we first briefly introduce the various concepts related to disease module on incomplete interactome, especially a quantity S_AB_, called module separation, as given in [4], to measure relationship between two disease modules A and B. Then we explain in detail our method of finding missing common genes for a given pair of diseases formulated as an optimization problem to minimize S_AB_.

### Disease module on Interactome and module separation

Interactome contains all protein-protein interactions in the cell, and can be conveniently represented as a graph (or network), in which proteins are represented as nodes and interaction between two proteins is represented as an edge connecting the two corresponding nodes. Reconstructing the interactome is a central task in systems biology, which studies the cell as a system in a holistic way instead of simple ensemble of isolated items. Due to the limitation of the current technology, interactome for most organisms, even model organisms, is incomplete, with missing nodes and edges. Nonetheless, the incomplete interactome can already provide valuable insights into many biological processes which cannot be obtained otherwise. In [4], it is shown how to uncover disease-disease relationships through the incomplete interactome. Diseases with genetic causes have been studied widely, often with a focus to identify the culprit gene only, to find that in many cases the cause cannot be attributed to a single gene; instead it is very common that multiple genes involving in multiple cellular processes may be at play. Without putting these pieces in a bigger context, it is difficult to fully understand the pathological mechanisms. Work in [4] presents a systematic study to uncover disease-disease relationships by mapping the associated genes onto the interactome.

As mentioned by [4], given a pair of diseases A and B, the genes known to be associated with them are put into two separate sets G_A_ and G_B_ respectively. Let graph G be the interactome, with node set V, and edge set E. Let map the genes in G_A_ and G_B_ onto G with two different colors, say, nodes in G corresponding to genes in G_A_ are colored red and nodes in G corresponding to genes in G_B_ are colored blue. For any shared gene, i.e., a gene is known to be associated to both disease A and disease B, then the corresponding node will be colored half red and half blue. Although all the red nodes are genes associated with disease A, indicating relatedness among them, they may not form a single connected component (or subgraph) of graph G of the interactome; often they form several connected components. This may be due to either incompleteness of the interactome (i.e., missing edges) or unknown associated genes, or a combination of both. However, if the connected components are too fragmented, say not significantly different from what can be formed by randomly mapped genes, then it is difficult to reliably infer useful relationships. So, in [4], the size of the largest connected component, as a percentage of the total number of genes associated to a disease, must be maintained beyond a threshold, which is set based on percolation theory and the data used in the study. And the largest connected component, meeting the size requirement, is then called module as representative for the disease. For example, multiple sclerosis (MS) has 69 known associated genes and the largest connected component, which is qualified as a module with a size of 11, and rheumatoid arthritis (RA) has 51 associated gene and the largest connected component, which is qualified as module with a size of 9.

**Fig. 1:**
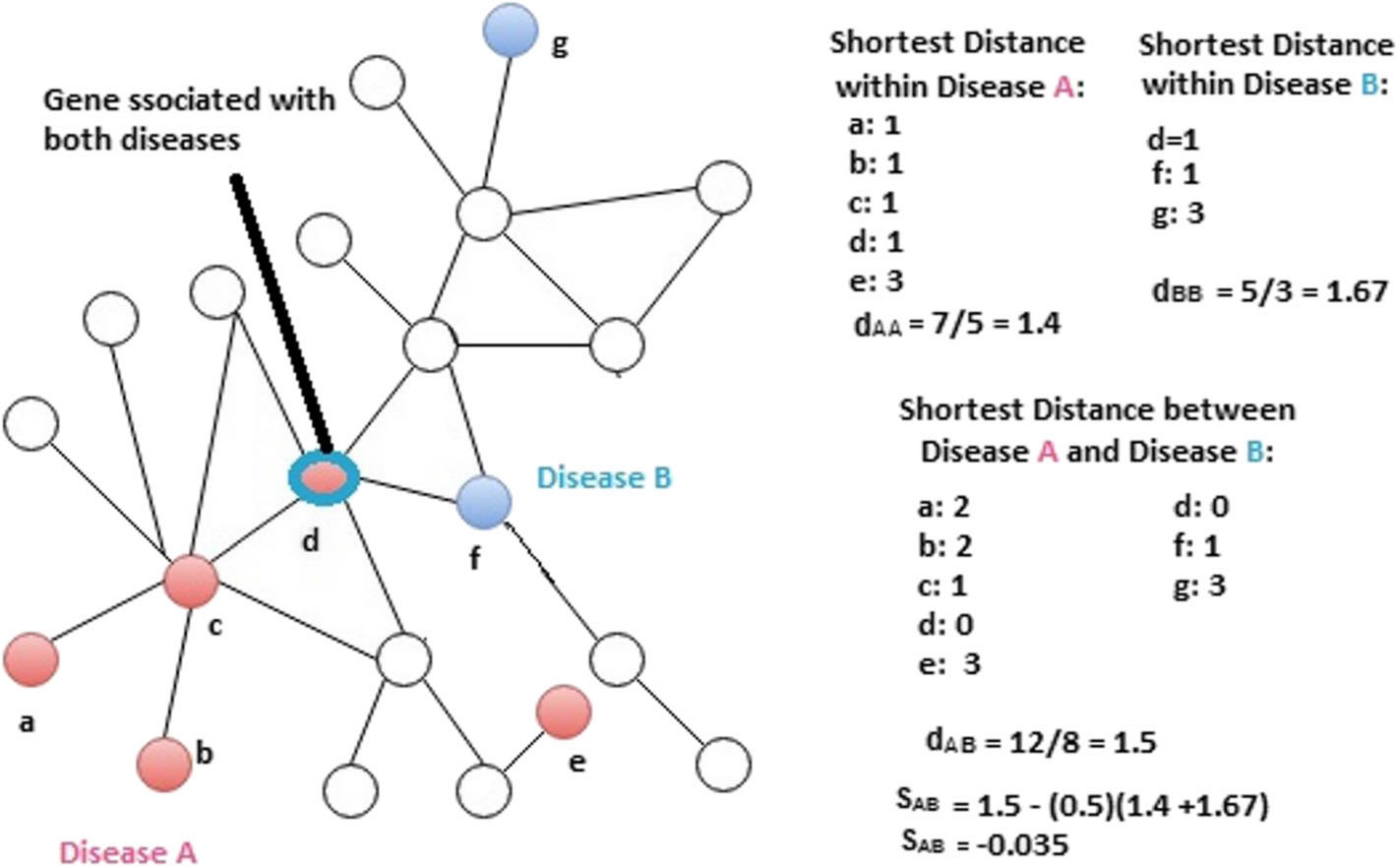
Illustration of network separation calculation

To uncover disease-disease relationships, a quantity called module separate S_AB_ is introduced as follows.

1 $$ {\mathit{\mathsf{s}}}_{\mathit{\mathsf{AB}}}\kern0.5em \equiv <{\mathit{\mathsf{d}}}_{\mathit{\mathsf{AB}}}>-\frac{<{\mathit{\mathsf{d}}}_{\mathit{\mathsf{AA}}}>+<{\mathit{\mathsf{d}}}_{\mathit{\mathsf{BB}}}>}{\mathsf{2}} $$

where <d_AB_> is the average of the shortest distance for each gene of disease A to reach a gene of disease B and vice versa, <d_AA_> is the average of the shortest distance for every gene in disease A to reach another gene in disease A, and <d_BB_> the average of the shortest distance for genes of disease B to reach another gene in disease B. Figure 1 shows how S_AB_ is computed for a toy example. More comprehensive results in [4] demonstrate that this network-based measurement of disease module separation is more indicative of pathological manifestations of disease pairs than simply measuring the overlap between the associated gene sets, such as Jaccard Index:

2 $$ \mathrm{J}=\kern0.5em \mid {\mathrm{G}}_{\mathrm{A}}\cap {\mathrm{G}}_{\mathrm{B}}\left|/\right|{\mathrm{G}}_{\mathrm{A}}\cup {\mathrm{G}}_{\mathrm{B}}\mid $$

It is reported in [4] that, when the disease history of 30 million individuals aged 65 and older is used to determine the relative risk RR of disease comorbidity for each disease pair, the relative risk drops from RR ≥ 10 for S_AB_ < 0 to the random expectation of RR ≈ 1 for S_AB_ > 0.

### Detection of missing shared genes

To further explore the predictive power of the disease module separation, we use it to tackle the incompleteness of the data. Specifically, for disease pairs that are known to share high comorbidity and therefore are expected to have a small, preferably negative, module separation, but instead have large positive S_AB_, we hypothesize that the discrepancy is due to some missing pieces of information, such as a missing shared gene, which if recovered should bring the two disease modules closer, i.e., to decrease S_AB_. Therefore, we formulate the detection of missing common genes between two disease modules as an optimization problem as follows.

3 $$ {\mathrm{x}}^{\ast }=\mathrm{argmin}\ \mathrm{SAB}\left[+\mathrm{x}\right] $$

$$ \mathrm{x}\in \left({\mathrm{G}}_{\mathrm{A}}\cup {\mathrm{G}}_{\mathrm{B}}\right)-\left({\mathrm{G}}_{\mathrm{A}}\cap {\mathrm{G}}_{\mathrm{B}}\right) $$

where x goes over genes distinctly associated to either disease A or disease B, and S_AB_[+x] is the module separation when x is added as a shared gene between disease A and B, and x* is the predicted missing shared gene which minimizes the module separation. The minimization can be achieved either by exhaustive search when the sets G_A_ and G_B_ are not very large or by some heuristics when the search space becomes huge. Note that, although Eq. (3) is formulated for finding a single (most probable) missing common gene, in practice, Eq. (3) can be applied sequentially multiple times for recovering multiple missing common genes. It is also worthwhile to note that the set of missing common genes recovered by using Eq. (3) iteratively one gene at a time may likely be different from a set of missing common genes should their candidacy as common gene be evaluated altogether, possibly due to the topology of the interactome and how these genes are located. So, if the number of missing common genes k is known, an alternative formulation of the optimization problem can be defined as follows.

4 $$ {\mathrm{X}}^{\ast }=\mathrm{argmin}\ {\mathrm{S}}_{\mathrm{AB}}\left[+\mathrm{X}\right] $$

$$ \mathrm{X}\in \left({\mathrm{G}}_{\mathrm{A}}\cup {\mathrm{G}}_{\mathrm{B}}\right)-\left({\mathrm{G}}_{\mathrm{A}}\cap {\mathrm{G}}_{\mathrm{B}}\right) $$

where X* is the optimal set of missing common genes, and X is any subset of size k from the genes that are distinctly associated with either disease A or disease B. This formulation, while theoretically sound and appealing, has two practical issues: a) the number of missing common genes k is not known a priori; and b) the increased computational complexity due to combinatorial in selecting k out n, where n = |G_A_ ⋃ G_B_| - |G_A_ ∩ G_B_|. Because of these issues, we only tested Eq. (4) for k = 2 and k = 3, while the results reported in the next section are mainly based on Eq. (3).

## Results

In this section, we tested our method for identifying missing genes with the data used in [4]. We first describe briefly the dataset, and then present the results which are evaluated using a cross validation scheme.

### Dataset

The data, including Human interactome, disease gene association, network properties of disease pairs and comorbidity data, was used in the study from [4] and was downloaded from their website. Comorbidity (RR score) for several diseases using medicare data from USA has been calculated by [8]. The dataset contains 913 disease modules with negative S AB value and known RR score. The comorbidity value ranges from 0 to 6497. Comorbidity value 1.0 or above is considered high [4]. Among the 605 disease modules, 148 of them have comorbidity value ranging from 3.0 to 6497, and only 25 disease pairs with RR score above 100. Most of the disease modules have pairwise RR score between 0 and 3.0.

While the method is ultimately aimed at finding de novo missing common genes between a disease pair, for evaluation purpose, the method is tested, in a cross-validation scheme, at recovering known common genes. Therefore, a disease pair must have common genes to be used in the test. It was found that, out of 913 disease pairs, there are 605 disease pairs that satisfy the requirement, and the remaining 308 disease modules, either do not have any common gene or have all the genes common and hence are removed from the test dataset.

### Cross-validation and performance

The cross-validation scheme is designed as follows. For a disease pairs A and B:

1. Randomly select multiple common genes and reserve them as positive test examples.
2. Randomly select multiple non-common genes from G_A_ and G_B_ respectively, and reserve them as negative test examples.
3. For each gene x in the test set, run the search algorithm as given in Eq. (3), and compute S_AB_[+x], the module separation when x is marked as shared, and x goes over all test examples associated with diseases A and B. Then compute prediction score s(x) = S_AB_ - S_AB_[+x].
4. Rank all the test examples x’s by s(x) in a descending order: the higher the score s(x), the higher that x is ranked and hence more likely to be a common gene. Receiver operating characteristic (ROC) score is computed by comparing the ranked list and the ground truth of the test examples.

Note that in the experiments reported below, 10 common genes, if available, were selected from G _A_ ∩ G_B_ and 10 uncommon genes were selected from (G_A_ ⋃ G_B_) – (G_A_ ∩ G_B_) for cross validation.

**Fig. 2:**
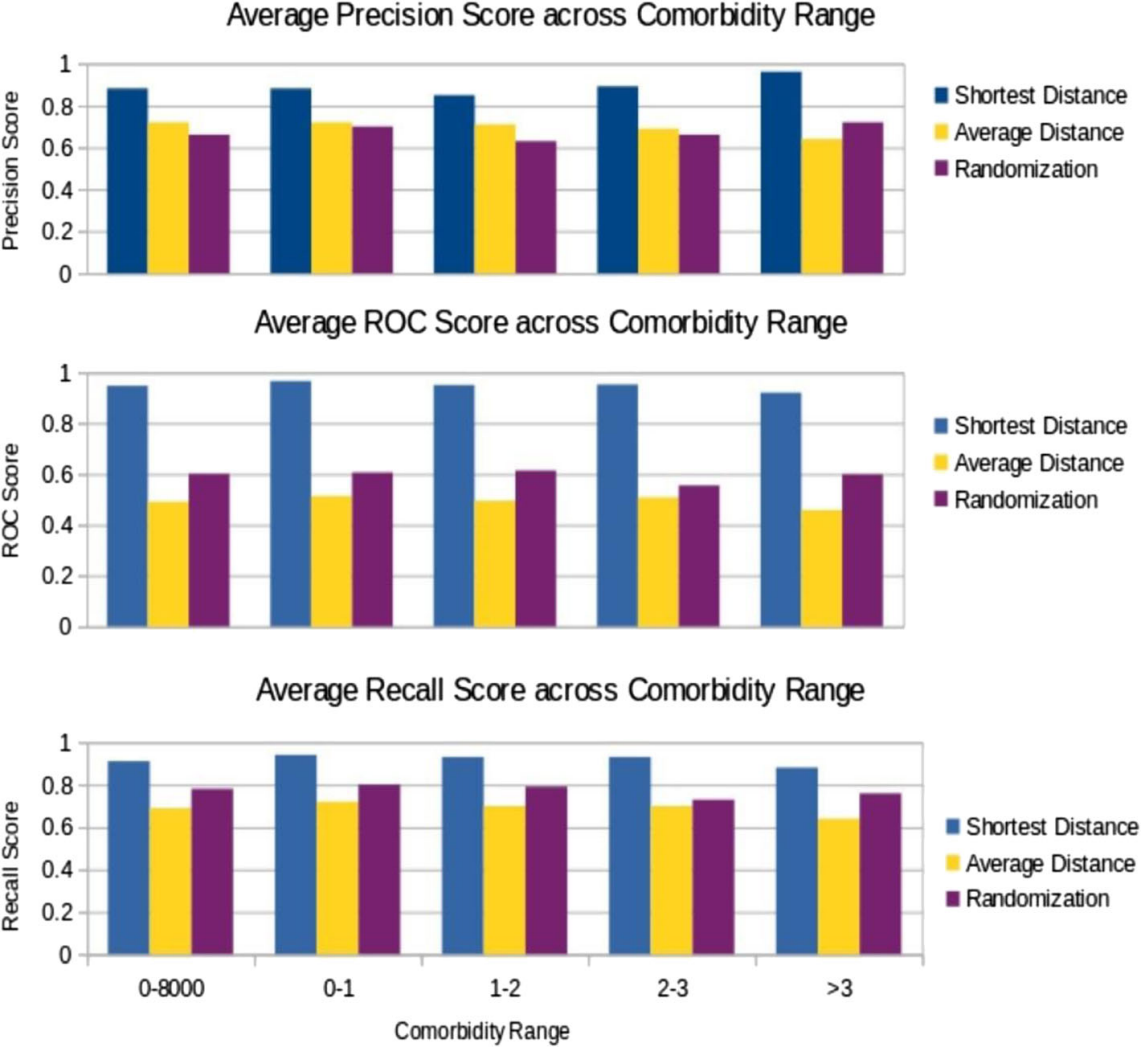
Bar chart for average ROC Score, average Precision and average Recall across comorbidity range

The performance is evaluated by using receiver operating characteristic (ROC) score. From the list of the test examples ranked by their prediction score s(x), ROC curve plots the true positive rate as the function of false positive rate when a threshold moves from the top to the bottom of the ranked list – test examples with prediction score larger than or equal to the threshold are predicted as positive and otherwise as negative. ROC score is the area under the curve of ROC curve and thus has a range of [0, 1], with 0.5 corresponding to a random classifier and higher score corresponding to better predictive power. The average ROC score for the whole dataset is 0.947, as reported in Table 1. When Eq. (4) is used in place of Eq. (3), the average ROC score is 0.976 and 0.979 for k = 2 and 3 respectively. This confirms that considering candidate missing common genes as a subset can indeed achieve better prediction as compared to considering candidate missing common genes individually, though the gain in performance seems to be tapering as the value of k increases. Table 1 also lists the average ROC score for several cases: a) disease pairs with comorbidity in [0,1], b) disease pairs with comorbidity in [1, 2], c) disease pairs with comorbidity in [2, 3], and d) disease pairs with comorbidity >3.0, with case e) being all pairs included. It can be seen clearly that high average ROC scores are achieved for all cases, with case a) achieving marginally the highest. This finding is noteworthy as it suggests that S_AB_ is a useful indicator across all range of relative risk (RR) value whereas in [4] strong correlation was observed between RR drops and S_AB_ switching from negative to positive. Precision and recall reported in Table 1 are computed using a threshold on prediction score s(x) which is set as suggested in [9]. Essentially, the threshold is set by using ROC curve on the test data to determine the highest peak point of ROC curve from the diagonal line, i.e., the prediction score of the test example that corresponds the peak point is used as the threshold. Average precision and recall are reported as 0.88 and 0.91 respectively for comorbid disease pairs using shortest distance as method to measure module separation. Figure 2 represents a graphical representation of the evaluation metrics (roc score, precision and recall) used for two methods for calculating module separation and when used for randomized data.

**Table 1:** Average ROC Scores with standard deviation, precision and recall for various comorbidity ranges

	Comorbidity Range				
	0-8000	0-1	1-2	2-3	>3
Number of Disease Pairs	605	133	248	76	148
Average ROC Score (Shortest Distance)	0.947	0.966	0.950	0.952	0.920
Stddev (Shortest Distance)	0.094	0.063	0.089	0.072	0.124
Average ROC Score (Average Distance)	0.491	0.513	0.495	0.508	0.458
Stdev (Average Distance)	0.279	0.279	0.288	0.269	0.269
Average ROC Score (Randomization)	0.601	0.606	0.614	0.555	0.599
Stedev (Randomization)	0.278	0.282	0.287	0.258	0.2468
Average Precision (Shortest Distance)	0.88	0.88	0.85	0.89	0.96
Stddev (Shortest Distance)	0.27	0.28	0.31	0.25	0.15
Average Precision (Average Distance)	0.72	0.72	0.71	0.69	0.64
Stdev (Average Distance)	0.311	0.31	0.32	0.33	0.30
Average Precision (Randomization)	0.66	0.70	0.63	0.66	0.72
Stedev (Randomization)	0.29	0.28	0.29	0.30	0.29
Average Recall (Shortest Distance)	0.91	0.94	0.93	0.93	0.88
Stddev (Shortest Distance)	0.13	0.11	0.13	0.09	0.16
Average Recall (Average Distance)	0.69	0.72	0.70	0.70	0.64
Stdev (Average Distance)	0.30	0.28	0.30	0.31	0.30
Average Recall (Randomization)	0.78	0.80	0.79	0.73	0.76
Stedev (Randomization)	0.26	0.25	0.26	0.26	0.25

**Fig. 3:**
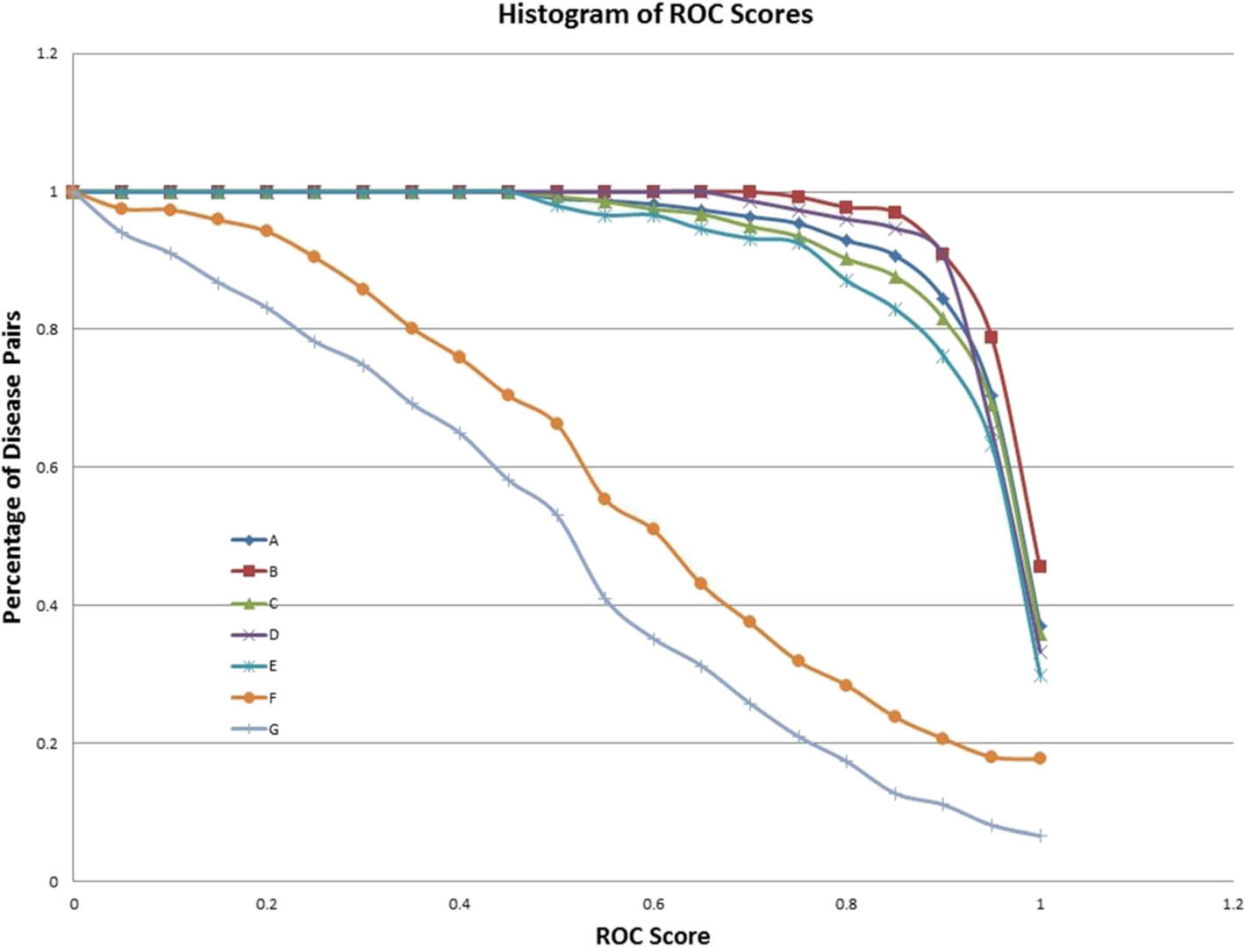
Histogram of ROC Scores. A: comorbidity range 0 ~ 1; B: comorbidity range 1~2; C: comorbidity range 2 ~3; D: comorbidity range > 3; E: comorbidity range 0 ~ 10,000; F: randomized common genes; G: SAB based on average distance

In addition to the average ROC scores, the histogram plot of ROC scores is shown in Fig. 3. In the histogram, a point in a curve shows in the vertical axis the percentage of disease pairs that have a performance greater or equal than ROC score given in the horizontal axis. It also shows the random ROC score in yellow color.

We further examined how the prediction performance is affected by the number of common genes, i.e., the size of the training set. Specifically, we grouped disease pairs based on the range of overlap between associated genes: i) 5 ~ 10 common genes, ii) 10 ~ 15, and iii) 15 or more common genes.

The effect of the size of training set and the range of RR on prediction performance is reported in Table 2, which lists the number of disease pairs achieving a given ROC score range for different groups under different RR range. For example, 42 pairs with 0~ 5 common genes and RR between 0 and 1.0 have received ROC score in the range (0.9, 1.0) The results show that as the number of common genes increases, the prediction performance in terms of distribution over various ranges is quite stable, with slight improvement, suggesting the method is robust under various conditions. In each case we had all the results above ROC score 0.5. And, more than 80% of the disease modules provide missing gene prediction ROC score between 0.9-1.

**Table 2:** Effect of the size of training set and the range of RR on prediction performance

ROC Score Range	Comorbidity Range				
	0-8000	0-1	1-2	2-3	>3
i) 0 ~ 5 Common Genes					
0.5-0.6	2	0	2	0	0
0.7-0.8	5	2	3	0	0
0.9-1.0	174	46	81	18	29
Total	181	48	86	18	29
ii) 5 ~ 10 Common Genes					
0.5-0.6	0	0	0	0	0
0.7-0.8	2	0	2	0	0
0.9-1.0	121	36	48	15	22
Total	123	36	50	15	22
iii) 10 - 15 Common Genes					
0.5-0.6	0	0	0	0	0
0.7-0.8	1	0	0	0	1
0.9-1.0	46	12	21	4	9
Total	47	12	21	4	10
iv) 15 or more Common Genes					
0.5-0.6	10	1	3	0	6
0.7-0.8	24	1	6	4	13
0.9-1.0	220	35	82	35	68
Total	254	37	91	39	87

## Discussion

It should be noted that the missing common gene problem, despite of its apparent importance, has not yet been addressed elsewhere in the literature to our best knowledge. Still, in order to get a sense how well the proposed method does in comparison to a baseline, we randomize the common genes for each disease pair. Specifically, for each disease pair, the set of common gene is replaced with the same number of genes randomly selected from the whole set of genes in the interactome. The rationale for doing so is to keep the count of common genes for each disease pair unchanged and also maintain the topology of interactome and the overall relative locations of the two diseases in the pair. When everything else was kept the same, it was found that the average ROC score dropped to 0.601 for the 605 disease pairs with their common genes randomized. The detailed results for different comorbidity ranges 250 with respect to the randomized baseline are listed in Table 1, and the histogram of ROC scores for the baseline is shown as plot F in Fig. 2.

For comparison, we also modify how the module separation is calculated. Specifically, instead of the shortest distances used in Eq. (1), we replaced <d_AB_> with the average distance for all distinct A -B gene pairs, <d_AA_> is the average distance for all gene pairs within disease module A, and <d_BB_> the average distance for all gene pairs within disease module B. Use this modified module separation, let’s call it all-pair-average based module separation S _<AB>_, we get an average ROC score 0.49 for all 605 disease pairs. The histogram of the ROC scores is shown in Fig. 2 as plot G. One plausible explanation of why the all-pair-average based module separation performs poorly is that the module separation has become much less sensitive to swapping a single gene x’s classification in Eq. (3) – from common gene to non-common gene and vice versa.

From comparison to the baseline of randomized data and an alternative definition of module separation, the results show that our proposed method performs very well, suggesting the optimization formulated in Eq. (3) as a viable solution to finding missing common genes for a given pair of diseases. Note that the predictive power is measured by ROC score, which does not require a pre-set threshold on the score s(x) when it is used for making prediction. Not requiring a pre-set threshold on the prediction score contributes to the popularity of ROC as a metric for assessing predictive power of a binary classifier: the ability to differentiating positive examples from negative examples when ranking on these examples by the prediction score. This is because in reality it is often difficult to set a priori threshold on the prediction score, although it can be set in certain ad hoc ways. In our situation, the score s(x), computed for each module separation sAB, depends on the interactome topology, the unknown number of missing genes and other factors, which makes it difficult to have a preset threshold for any give disease pair, least to say a common threshold for all disease pairs. Even if we normalize the score s(x) as s(x) = (S_AB_ – S_AB_[+x]) / S_AB_ it is unlikely that a threshold set for one disease pair (e.g., by using the method cited in Ref [9]) would be the same for another disease pair, because different disease pairs can have their genes residing on different locations of the network (and hence having different network topologies) and can have different number of missing common genes. So, for practical use of our method, we envision that, for any pair of diseases with high comorbidity yet a large module separation, biologists would suspect some common genes are missing and then use our method to suggest a short list of candidates (i.e., these with top ranking score s(x)) for further investigation.

In this work, we used brute force to search all genes associated to the disease pair, as our focus is on the viability of using module separation to detect missing common genes not on the speed. In the dataset, we used for this study, the average number of genes in a disease pair is 168 and it takes 2 min 43 s to search all genes in the disease pair for putative common genes on a desktop computer: 2.90Ghz intel core i7, 8.00Gb memory. While it is desirable as a future work to find a faster heuristic algorithm for search as the number of genes increase, the brute force approach seems to be acceptable for typical cases.

## Conclusions

In this work, we developed a novel method to predict missing common genes for a given disease pairs. The method formulates the task as an optimization problem of minimizing network based module separation for subgraphs formed by associated genes on the interactome, with the hypothesis that correctly identified missing common genes would bring the two-module closer. The results of cross-validation from a benchmark dataset of more than 600 disease pairs show high prediction accuracy on average, measured as ROC score. The method provides a useful tool to infer better understanding of disease- disease interaction in terms of related genes. While the method is tested in cross-validation mode in this study, it can be easily deployed to predict de novo *m*issing genes, i.e., those genes that are not associated with any disease but have an impact on the phenotype of both diseases. It is worthwhile to note that the results reported in this study are based on incomplete Human interactome – protein interactions that exist but have not be detected by experiments and reported in literature, and thus are referred to as missing edges in the protein-protein interaction network. Therefore, the accuracy for missing gene prediction may change, likely for higher, as the interactome becomes more complete. In fact, as an effort to address the challenge presented by missing data, in future work this method could be extended for predicting missing edges in an incomplete interactome as well.
